# Substituted *trans*-stilbenes can inhibit or enhance the TPA-induced up-regulation of activator protein-1

**DOI:** 10.1186/1471-2210-8-19

**Published:** 2008-11-10

**Authors:** Lorraine M Deck, Lucy A Hunsaker, Amanda M Gonzales, Robert A Orlando, David L Vander Jagt

**Affiliations:** 1 Department of Chemistry, University of New Mexico, Albuquerque, NM 87131, USA; 2 Department of Biochemistry and Molecular Biology, University of New Mexico School of Medicine, Albuquerque, NM 87131, USA

## Abstract

**Background:**

The activator protein-1 (AP-1) family of transcription factors contributes to regulation of numerous genes involved in proliferation, apoptosis, and tumorigenesis. A wide array of stimuli can activate AP-1, including pro-inflammatory cytokines, growth factors, tumor promoters and stress. Numerous plant polyphenols have been shown to inhibit the activation of AP-1, which often is ascribed to the anti-oxidant properties of these natural products.

**Methods:**

In the present study, a library of substituted *trans-*stilbenes, including polyphenols, was screened for activity against the TPA-induced activation of AP-1 using the Panomics AP-1 Reporter 293 Stable Cell Line, which is designed for screening potential inhibitors or activators.

**Results:**

Several *trans*-stilbenes were identified that inhibit TPA-induced activation of AP-1, with IC_50 _values as low as 0.5 μM. Moreover, some other *trans*-stilbenes were able to enhance the effects of TPA 2 to 3-fold. Many of the *trans*-stilbenes identified as inhibitors or enhancers are devoid of anti-oxidant properties.

**Conclusion:**

The ability of *trans*-stilbenes to inhibit or enhance the effects of TPA does not depend upon their anti-oxidant properties.

## Background

Activator protein-1 (AP-1) transcription factors are homo- or heterodimers of members of the Jun (c-Jun, JunB, JunC), Fos (c-Fos, FosB, Fra-1, Fra-2), ATF (ATF2, ATF3, B-ATF, JDP1, JDP2) and Maf (c-Maf, MafB, MafA, MafG/F/K, Nrl) families of proteins, all of which are bZIP proteins. AP-1 dimers contribute to regulation of many cellular processes including proliferation, cell cycle regulation, differentiation, and apoptosis [1-6]. Active AP-1 dimers can bind to TPA-responsive elements (TREs) in the promoters of AP-1 responsive genes. AP-1 binding to TREs also is induced by growth factors, cytokines and oncoproteins, leading to the general view that activation of AP-1 is oncogenic by contributing to proliferation, survival and transformation of cells. Several AP-1 proteins, including c-Jun and c-Fos, can transform cells in culture [7-9]. Development of inhibitors of activation of AP-1 may be a promising approach to development of new anti-cancer therapeutics [10,11]. However, certain AP-1 dimers can be anti-oncogenic [4,12,13]. Whether or not AP-1 is oncogenic depends upon cell type, genetic background, nature of the stimulus and state of differentiation. AP-1 is also an important family of transcription factors involved in gene regulation in inflammation [14].

Activation of AP-1 can be inhibited by numerous natural product polyphenols such as resveratrol, curcumin, epigallocatechin gallate and theaflavins [6,15,16]. For example, resveratrol suppressed TNF-induced activation of AP-1 in a variety of cells through inhibition of activation of MAP kinases [17]. Resveratrol inhibited the TPA-induced expression of c-Fos and c-Jun in mouse skin, also by inhibiting MAP kinases [18,19]. In other studies, resveratrol inhibited anchorage-independent growth of melanoma cells by altering the dimeric composition of AP-1[20].

Resveratrol is a stilbene derivative. Both *cis*- and *trans-*resveratrol exist as natural products and both are biologically active [21]. It is often assumed that the biological properties of resveratrol and other natural product polyphenols are derived from their anti-oxidant properties. In the present study, a library of substituted *trans-*stilbenes was examined for activity as inhibitors or activators of the TPA-induced activation of AP-1 in the Panomics AP-1 Reporter 293 Stable Cell Line, which is the HEK293 cell transfected with an AP-1-dependent luciferase construct. We report here that substituted *trans*-stilbenes devoid of anti-oxidant activity can function as inhibitors of the TPA-induced activation of AP-1. Moreover, some *trans-*stilbenes can function as enhancers of the TPA-induced activation of AP-1.

## Methods

### Synthesis of trans-stilbenes

The synthesis of a library of substituted *trans*-stilbenes was reported previously [22].

### Assay of the anti-oxidant activities of trans-stilbenes

The anti-oxidant activities of the library of substituted *trans*-stilbenes were determined using two standard assays [23]. The *total radical-trapping anti-oxidant parameter *(TRAP) assay measures the ability of an analog to react with the pre-formed radical monocation of 2,2'-azinobis-(3-ethylbenzothiazoline-6-sulfonic acid) (ABTS^.+^) [24]. ABTS was reacted with potassium persulfate in the dark, overnight, to generate the colored ABTS^.+ ^radical cation, which has an absorption maximum at 734 nm. The activities of the *trans*-stilbenes were determined by their abilities to quench the color of the radical cation. The *ferric reducing/anti-oxidant power *(FRAP) assay measures the ability of an analog to reduce a ferric tripyridyltriazine complex [25]. The ferric complex of 2,4,6-tripyridyl-*s*-triazine was prepared at acidic pH, and the anti-oxidant activities of the *trans*-stilbenes were determined by their abilities to reduce the ferric complex to the ferrous complex, monitored by formation of ferrous complex at 593 nm. In both colorimetric assays, the vitamin E analog Trolox was used as a control.

### Culturing of AP-1 reporter cells

An AP-1 reporter stable cell line derived from human 293T embryonic kidney cells transfected with a luciferase reporter construct containing three AP-1 binding sites in the promoter (293T/AP-1-luc, Panomics, Inc., Redwood City, CA, USA) was grown in a humidified atmosphere at 37°C in 5% CO_2_/95% air. The cells were maintained in Dulbecco's Modified Eagle's Medium (DMEM – high glucose containing 4 mM glutamine) supplemented with 10% fetal bovine serum (FBS), 1 mM sodium pyruvate, 100 units/ml penicillin, 100 μg/ml streptomycin and 100 μg/ml hygromycin (Gibco/Invitrogen, Carlsbad, CA, USA) to maintain cell selection.

### Assay of the activities of substituted trans-stilbeness as inhibitors of the TPA-induced activation of AP-1

One day prior to treatment, the 293T/AP-1-luc cells were plated into 24-well cell culture plates (Costar, Cambridge, MA, USA) in the above media without hygromycin. The following day, the cells, which were at approximately 60% confluency, were fed fresh media with or without TPA, 10 ng/ml, (Calbiochem, USA) and immediately treated with resveratrol or a *trans-*stilbene prepared in DMSO stock solutions. The cells were placed again in a humidified atmosphere at 37°C in 5% CO_2_/95% air for 24 hours. Plate wells were gently washed with phosphate buffered saline, pH 7.4, and lysed with 1× passive lysis buffer (Promega, Madison, WI). The subsequent lysates were analyzed with the Luciferase Assay System (Promega, USA) utilizing a TD-20/20 luminometer (Turner Designs, Sunnyvale, CA, USA). The firefly luciferase relative light units were normalized to protein (mg/ml) with BCA™ Protein Assay Kit (Pierce, Rockford, IL, USA) and standardized to percent of control (TPA control).

### Culturing of murine macrophage cells

Murine macrophage cells (BV-2) were kindly provided by Dr. Paul M. Stemmer (Institute of Environmental Health Sciences, Wayne State University, Detroit, MI, USA). Cells were cultured in RPMI-1640 (Cellgro, Herndon, VA, USA) supplemented with 10% FBS, 1 mM sodium pyruvate, 2 mM L-glutamine, 100 μg/ml streptomycin sulfate and 100 units/ml penicillin. Cells were grown on culture plates, pre-treated with 1% gelatin for 30 min at 37°C, and passaged twice weekly.

### Measurement of secreted prostaglandin E2 (PGE2) levels

BV-2 cells were incubated without or with 10 ng/ml TPA. Those cells that were treated with TPA were incubated in parallel with resveratrol or analogs **1**, **29**, **46**or **48**at the indicated concentrations or with vehicle alone (dimethylsulfoxide, 0.1% final concentration). After 24 h incubation, culture media was removed and secreted PGE2 levels were quantified by competitive ELISA according to directions provided by the manufacturer (R&D Systems, Inc., Minneapolis, MN, USA).

### Statistics

**Figure 1:**
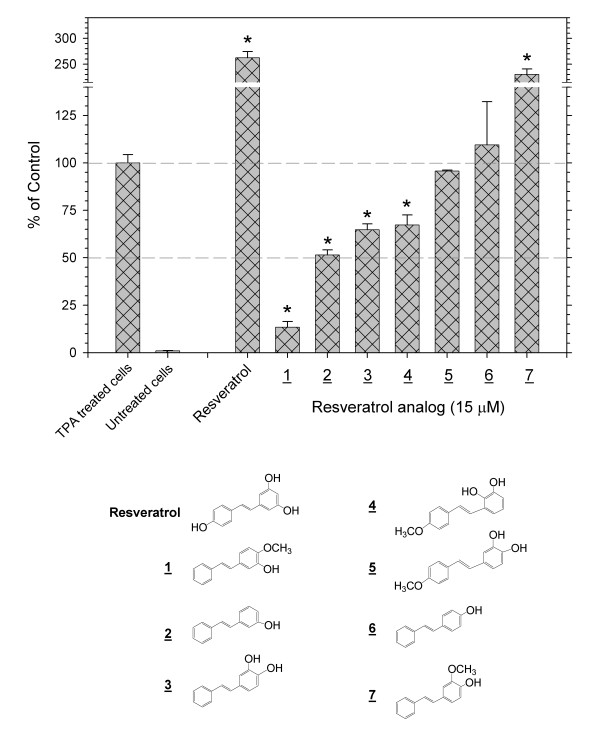
**Inhibition or enhancement of the TPA-induced activation of AP-1 by resveratrol and phenolic *trans*-stilbenes**. Screening with the 293T/AP-1-luc cell for the effects of resveratrol and phenolic *trans*-stilbenes on TPA-induced activation of AP-1 demonstrated that resveratrol and **7**were enhancers, **1**, **2**, **3**and **4**were inhibitors, and **5**and **6**were inactive. These phenolic compounds retain anti-oxidant activity. Each value represents the average of triplicate points. Error bars show standard deviations. The * notation indicates p < 0.01.

**Figure 2:**
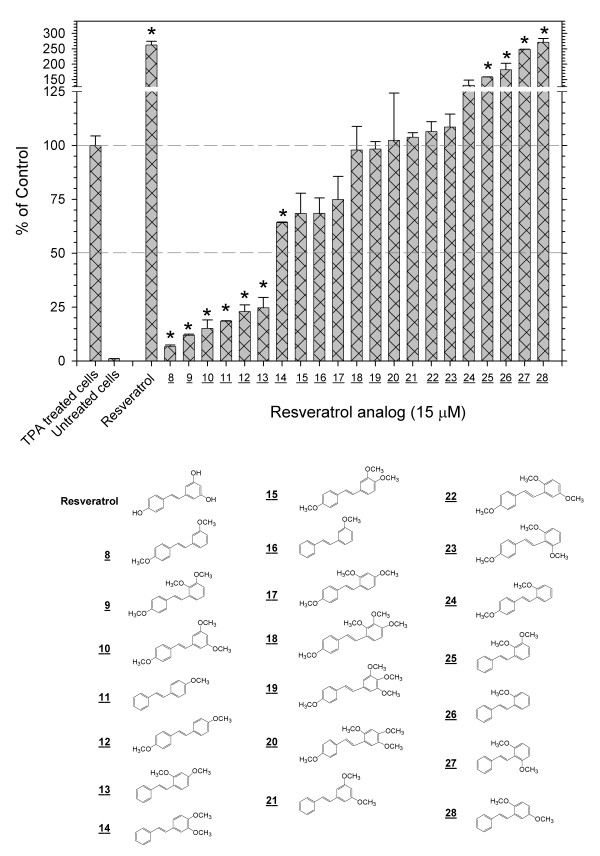
**Inhibition or enhancement of the TPA-induced activation of AP-1 by methoxy-substituted *trans*-stilbenes**. Screening with the 293/AP-1-luc cell of methoxy-substituted *trans*-stilbenes, which do not retain anti-oxidant activity, demonstrated that some derivatives inhibit, some enhance and others have no effects on the TPA-induced activation of AP-1. The * notation indicates p < 0.01.

**Figure 3:**
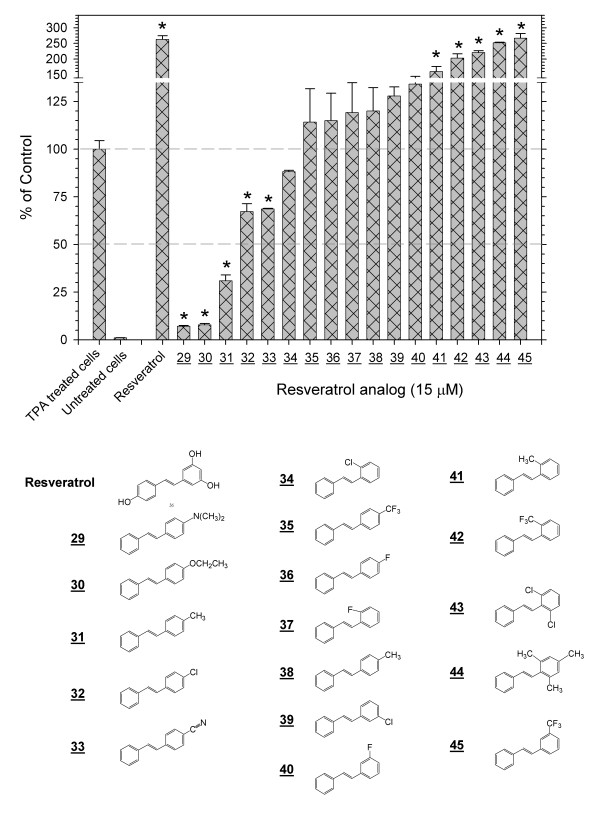
**Inhibition or enhancement of the TPA-induced activation of AP-1 by substituted *trans*-stilbenes devoid of hydroxy or methoxy functional groups**. Screening with the 293/AP-1-luc cell of a range of substituted *trans*-stilbenes, which do not have hydroxyl or methoxy functional groups, demonstrated that some derivatives inhibit, some enhance and others have no effects on the TPA-induced activation of AP-1. The * notation indicates p < 0.01.

**Figure 4:**
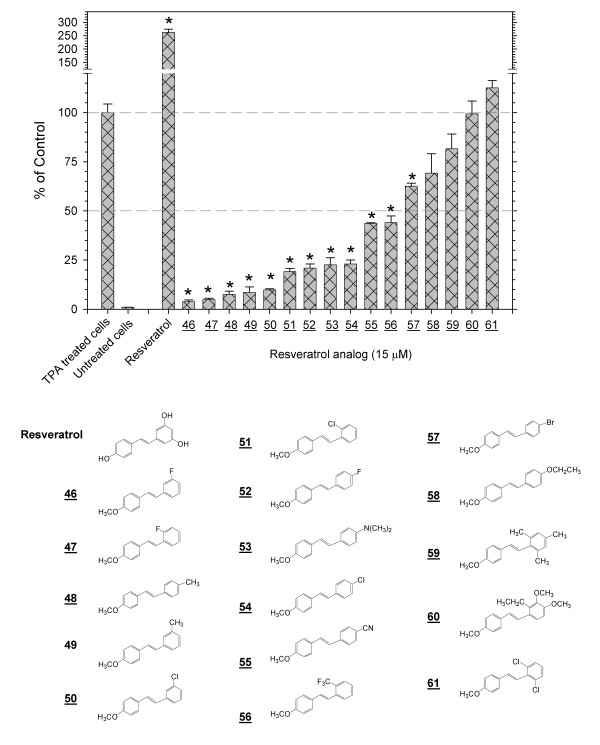
**Inhibition of the TPA-induced activation of AP-1 by *para*-methoxy-substituted trans-stilbenes**. Screening with the 293/AP-1-luc cell of a range of substituted *trans*-stilbenes, all of which have a *para*-methoxy functional group on one ring, demonstrated numerous inhibitors but no enhancers of the TPA-induced activation of AP-1. Analog **46**, with an IC_50 _= 0.5 μM, was the most potent inhibitor observed in this study. The * notation indicates p < 0.01.

**Figure 5:**
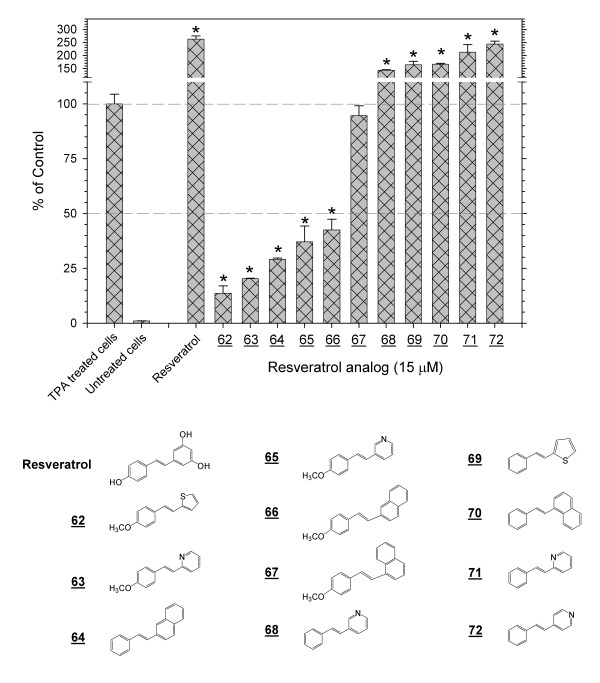
**Inhibition or enhancement of the TPA-induced activation of AP-1 by analogs of *trans*-stilbenes**. Screening with the 293/AP-1-luc cell of a range of analogs of stilbenes that retain the *trans *double bond demonstrated that inhibitors or enhancers of the TPA-induced activation of AP-1 can deviate considerably from the *trans-*stilbene scaffold. The * notation indicates p < 0.01.

**Figure 6:**
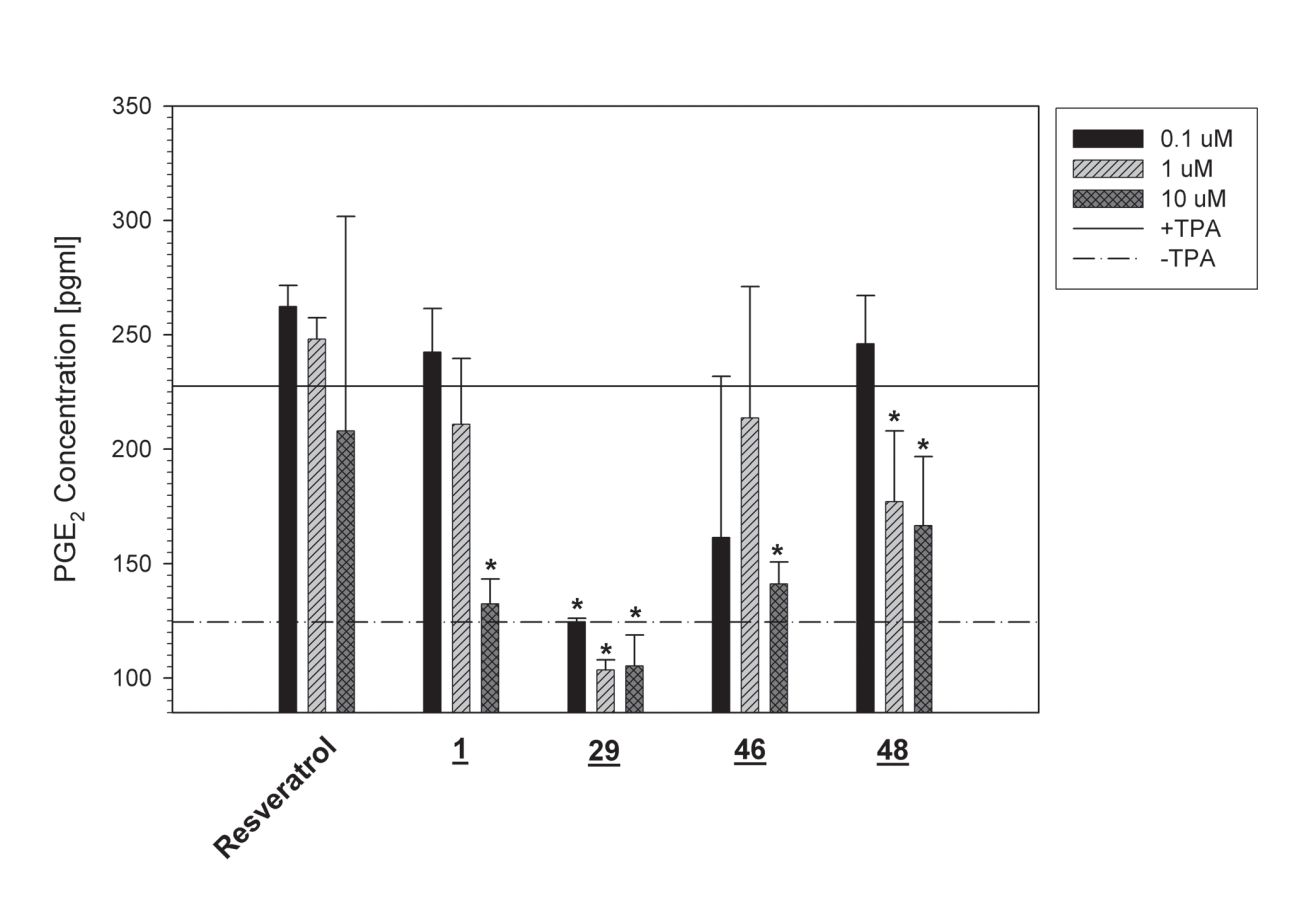
**Secreted PGE2 levels after treatment of TPA-activated murine macrophage cells with substituted *trans*-stilbenes**. BV-2 cells were activated with TPA and co-incubated with resveratrol, **1**, **29**, **46**, or **48**. Secreted PGE2 levels were quantified by competitive ELISA. Basal levels of secreted PGE2 were established using non-stimulated BV-2 cells (125 pg/ml). Maximal PGE2 levels were determined by treating BV-2 cells with TPA alone and no inhibitor (230 pg/ml). Analog **29**was the most potent inhibitor of PGE2 secretion. The * notation indicates p < 0.05.

Error bars in figures 1, 2, 3, 4, 5, 6 represent standard deviations from triplicate measurements. The ± values in Tables 1 and 2 indicate standard deviations from triplicate measures of IC_50 _from dose-response curves.

**Table 1:** IC_50 _values of substituted *trans*-stilbenes as inhibitors of the TPA-induced activation of AP-1

Number	Structure	IC_50 _(μM)	TRAP	FRAP
**1**		**0.8 ± 0.05**	+	+
**8**		**0.7 ± 0.04**	-	-
**9**		**2.4 ± 0.7**	-	-
**10**		**3.8 ± 1.1**	-	-
**11**		**1.3 ± 0.03**	-	+
**29**		**1.1 ± 0.1**	+	+
**30**		**0.8 ± 0.1**	-	+
**31**		**1.6 ± 0.6**	-	+
**46**		**0.5 ± 0.1**	-	-
**47**		**0.8 ± 0.1**	-	-
**48**		**0.8 ± 0.03**	-	-
**49**		**1.0 ± 0.12**	-	-
**50**		**0.8 ± 0.15**	-	-
**62**		**2.5 ± 0.5**	-	-
**63**		**2.1 ± 0.4**	-	-

Anti-oxidant Activity

**Table 2:** Anti-oxidant activities of *trans*-stilbene that enhance the TPA-induced activation of AP-1

Number	Structure	% Enhancement	TRAP	FRAP
**Resveratrol**		**262 ± 12**	+	+
**7**		**230 ± 11**	+	+
**25**		**158 ± 0.1**	-	-
**26**		**182 ± 21**	-	-
**27**		**248 ± 0.5**	-	-
**28**		**172 ± 12**	-	-
**41**		**160 ± 16**	-	-
**42**		**203 ± 14**	-	-
**43**		**220 ± 6**	-	-
**44**		**251 ± 2**	-	+
**45**		**267 ± 15**	-	-
**68**		**143 ± 3**	-	-
**69**		**164 ± 13**	-	-
**70**		**167 ± 4**	-	+
**71**		**213 ± 29**	-	-
**72**		**243 ± 12**	-	-

Anti-oxidant Activity

## Results

### Inhibition or enhancement of the TPA-induced activation of AP-1 by resveratrol and phenolic trans-stilbenes

Resveratrol and 7 *trans-*stilbenes that contained one or more phenolic substituents were compared for their effects on the TPA-induced activation of AP-1 in an initial screening at 15 μM concentration of the compounds (Figure 1). Some of the compounds were strong inhibitors of the activation of AP-1. Analog **1**, which contains hydroxy and methoxy functional groups ortho to each other on one of the two aromatic rings, was the strongest inhibitor. From dose-response measurements, the IC_50 _for **1**was 0.8 μM (Table 1). By comparison, **7**, which is isomeric with **1**, enhanced the TPA-induced activation of AP-1 2.5-fold. Clearly, small changes in structure can dictate whether a *trans-*stilbene inhibits or enhances the effects of TPA. Surprisingly, in this screening assay, resveratrol enhanced the effects of TPA almost 3-fold. Other compounds in this series, such as **5**and **6**, had no effect. All of the *trans*-stilbenes in Figure 1 retain anti-oxidant activity both in the TRAP and FRAP assays.

### Inhibition or enhancement of the TPA-induced activation of AP-1 by methoxy-substituted trans-stilbenes

A series of 21 methoxy-substituted *trans-*stilbenes containing one to four methoxy functional groups was screened at 15 μM, as above (Figure 2). A wide range of effects was observed. Some of these *trans-*stilbenes, such as **8**, **9**, **10**and **11**, were strong inhibitors of the TPA-induced activation of AP-1 with IC_50 _values in the range 0.7 to 3.8 μM (Table 1). Others, such as **26**, **27**and **28**, gave 2 to 3-fold enhancements, comparable to resveratrol. Analogs **18**, **19**, **20**and **21**were inactive. Methoxy-substituted *trans*-stilbenes, including **8**, **9**and **10**, which were among the most potent inhibitors, were generally devoid of anti-oxidant activity in the TRAP and FRAP assays, indicating that anti-oxidant activity is not essential.

### Inhibition or enhancement of the TPA-induced activation of AP-1 by substituted trans-stilbenes devoid of hydroxy or methoxy functional groups

A series of 17 substituted *trans*-stilbenes that did not contain hydroxy or methoxy functional groups was screened at 15 μM as above (Figure 3). Several of these, such as **44**and **45**, which contained methyl of trifluoromethyl groups on one of the aromatic rings, were effective enhancers of the TPA-induced activation of AP-1, comparable to resveratrol. Several others, such as **29**, **30**and **31**, which contained a single dimethylamino, ethoxy or methyl functional group, were strong inhibitors (Table 1). Many of the compounds in this series were weak inhibitors or weak activators.

### Inhibition or enhancement of the TPA-induced activation of AP-1 by para-methoxy-substituted trans-stilbenes

A series of 16 substituted *trans-*stilbenes that contained a *para*-methoxy substituent on one ring and a variety of substituents on the second ring was screened at 15 μM (Figure 4). In this series, many compounds were strong inhibitors of the TPA-induced activation of AP-1, such as **46**, **47**, **48**, **49**and **51**(Table 1). Others were weak inhibitors or showed no activity. None of the *trans*-stilbenes in this group enhanced the TPA-induced activation of AP-1. Thus minor changes in the nature or location of a single substituent markedly affects activity. Analog **46**, with an IC_50 _= 0.5 μM, was the most potent inhibitor observed in this study (Table 1).

### Inhibition or enhancement of the TPA-induced activation of AP-1 by analogs of trans-stilbenes

A series of 11 analogs of *trans-*stilbenes was evaluated. This group retained a *trans *double bond as in *trans-*stilbene. One of the aromatic benzene rings was replaced by a different ring. As with the other series, a range of activities was observed (Figure 5). Some compounds, such as **62**and **63**, were strong inhibitors (Table 1) while others, such as **71**and **72**, gave enhancements comparable to resveratrol. Clearly, structures of inhibitors or enhancers of the TPA-induced activation of AP-1 can differ considerable from a strict *trans-*stilbene scaffold.

### Effect of substituted trans-stilbenes on secreted prostaglandin E2 (PGE2) levels

To determine whether the AP-1 inhibitory effects of substituted *trans-*stilbenes extend beyond the 293/AP-1-luc reporter cell line used for screening, several compounds were selected from Table 1 and examined for capacity to reduce levels of secreted PGE2 by a TPA-activated murine macrophage cell line (BV-2). PGE2 is a potent inflammatory mediator and product of cyclooxygenase-2 (COX-2) activity. Since COX-2 activity is an important target for traditional anti-inflammatory reagents, such as non-steroidal anti-inflammatory drugs, quantifying secreted PGE2 can provide a direct measurement of COX-2 expression levels. When BV-2 cells were stimulated with TPA, levels of secreted PGE2 were elevated by ~2-fold (Figure 6). All compounds tested, except resveratrol, demonstrated a dose-dependent inhibitory effect on levels of PGE2. Analog **29**was the most potent inhibitor as 0.1 μM was sufficient to reduce PGE2 levels to those measured for unstimulated cells. Analogs **1**and **46**were also able to reduce PGE2 levels to baseline, but required doses of 10 μM, making **29**~100-fold more potent that these other *trans-*stilbenes. It was assumed that the measurement of secreted PGE2 in response to treatment with resveratrol or analogs reflects the effects of these compounds on COX-2 gene expression. It is possible that the compounds are inhibitors of COX-2 enzyme. However, we demonstrated previously in similar studies that focused on NF-κB-dependent expression of COX-2 in response to LPS stimulation that the inhibitory effects of resveratrol and analogs were at the level of gene expression as determined by mRNA measurements [22].

### Anti-oxidant activities of enhancers and inhibitors of TPA-induced activation of AP-1

As mentioned above and shown in Table 1, *trans*-stilbenes that inhibit the TPA-induced activation of AP-1 need not retain anti-oxidant activity, as determined by the TRAP and FRAP assays. This same property was observed with enhancers of the TPA-induced activation of AP-1. As shown in Table 2, most of the compounds that enhance activity are devoid of antioxidant activity.

## Discussion

The ability of resveratrol to inhibit the stress-induced activation of AP-1 has been reported in a number of studies. In addition to suppressing TNF-induced activation of AP-1 in a variety of cells, inhibiting TPA-induced expression of c-Fos and c-Jun in mouse skin, and inhibiting anchorage-independent growth of melanoma cells [18-20], resveratrol suppressed TPA-induced expression of MMP-9 by inhibition of AP-1 activation through c-Jun N-terminal kinase (JNK) and PKC-delta pathways [26] and protected against 4-hydroxynonenal-induced apoptosis by blocking AP-1 signaling through JNK [27]. These and other studies of the effects of resveratrol generally support the idea that resveratrol prevents AP-1 activation by inhibiting upstream kinases. However, in other studies, resveratrol and closely related polyphenols, such as isorhapontigenin, inhibited AP-1 activation through suppression of ROS-induced activation of MAP kinases by preventing the accumulation of ROS [28]. In some studies, resveratrol induced rather than inhibited protein expression; resveratrol induced COX-2 expression and nuclear accumulation in breast cancer cells through activation of MAP kinases and AP-1 [29]. In HT-29 cancer cells transfected with an AP-1 reporter, resveratrol enhanced AP-1 activity induced by TPA, similar to the present study [30]. It is apparent, therefore, that the effects of resveratrol and related compounds with respect to AP-1 signaling are cell specific, although generally resulting in inhibition of the activation of AP-1.

These multiple studies of the biological activities of resveratrol generally do not address the question whether anti-oxidant activity of resveratrol is required for the reported biological activity. AP-1 is one of a number of redox-sensitive transcription factors. AP-1 activity is regulated either directly or indirectly through reversible oxidation-reduction of critical cysteine residues [31,32]. c-Jun and c-fos have single conserved cysteine residues in their DNA-binding domains that undergo reversible redox reactions that alter their DNA-binding properties [33]. There also are a number of AP-1-associated proteins, such as thioredoxin, jun activation domain-binding protein 1 (Jab1), and redox factor-1 (Ref-1), that contribute to regulation of the redox state of AP-1 [34,35]. The present study addressed this question through evaluation of the effects of *trans*-stilbenes, many of which did not retain anti-oxidant activity, on AP-1 expression in response to TPA. Numerous *trans*-stilbenes devoid of anti-oxidant activity in the TRAP and FRAP assays were able to suppress the TPA-induced activation of AP-1, including many of the more potent compounds (Table 1). Clearly, anti-oxidant activity is not essential for the ability of these *trans*-stilbenes to affect activation of AP-1.

The question of the role of anti-oxidant activity in those substituted *trans*-stilbenes that enhance the TPA-induced activation of AP-1 was also addressed (Table 2). As is the case with *trans*-stilbenes that inhibit the TPA-induced activation of AP-1, there is no required role for anti-oxidant activity. In fact, most of the compounds that either suppressed or enhanced TPA-induced AP-1 activity in this study are devoid of anti-oxidant activity.

This study did not address the question of the actual targets of resveratrol and other *trans*-stilbenes that result in either inhibition or enhancement of AP-1 activity in response to TPA-induced activation of the 293T/AP-1-luc cells. As mentioned above, the reports in the literature on AP-1 activation suggest that numerous upstream targets, especially protein kinases, might be involved in signaling pathways that control the expression of AP-1. Interestingly, a number of the most active analogs identified in the current study (Table 1) were also identified in our earlier study of the inhibition of expression of pro-inflammatory transcription factor NF-κB in response to exposure to LPS [22], suggesting that there may be common targets for both the AP-1 and NF-κB pathways that are inhibited by some of these *trans-*stilbenes. These targets remain to be identified.

The possibility should be considered that the substituted *trans*-stilbenes used in this study, especially those that inhibit both AP-1 and NF-κB pathways, may target multiple sites in the AP-1 and NF-κB pathways and that interactions between the AP-1 and NF-κB pathways may provide additional complexity that contributes to cell specific effects of these compounds. Recent studies support the complexity of the interactions between AP-1 and NF-κB. It was reported recently that the natural product curcumin, which like resveratrol is a polyphenolic compounds with multiple biological activities, is able to reduce expression of matrix metalloproteinases (MMPs) in breast cancer cells [36]. This was shown to involve both NF-κB and AP-1 pathways; silencing of the p65 subunit of NF-κB was sufficient to downregulate expression of AP-1 (c-jun) and MMPs, which suggests signaling was from the NF-κB pathway to the AP-1 pathway. It is often assumed that the site(s) of regulation of signaling through these pathways involves one or more of the numerous upstream kinases, such as MAP kinases, that control the activity of AP-1 and NF-κB. However, another recent study demonstrated that AP-1 and NF-κB can form nuclear complexes with each other [37].

The results shown in figure 6 for the effects of resveratrol and several of the *trans*-stilbenes on expression of COX-2, as determined by measurement of secreted PGE2, raise a number of issues. Resveratrol did not significantly inhibit the TPA-induced activation of COX-2 (figure 6) in a macrophage cell line. In a number of studies, COX-2 induction by TPA in various cell types has been shown to involve signaling through AP-1 [38,39] and sometimes to involve both the AP-1 and NF-κB pathways [40]. In addition, resveratrol is reported to inhibit TPA-induced COX-2 expression in mouse skin [19]. However, resveratrol has been reported to induce COX-2 expression and nuclear accumulation in breast cancer cells through an AP-1-dependent pathway [29]. Thus, the effects of resveratrol are context-dependent. This raises the question of the usefulness of reporter assays for screening compounds that may inhibit signaling pathways. It may be preferable to screen in a disease-specific manner by selecting cells that are appropriate models for a given disease and conducting phenotypic screens in which the appropriate disease-related genes are monitored, such as using macrophage cells to screen libraries for abilities to inhibit stress-induced induction of pro-inflammatory genes and using breast epithelial cells to screen for abilities to inhibit stress-induced induction of pro-growth, pro-metastatic genes.

The data in figure 6 also demonstrate that, for the few compounds selected from Table 1, quantitatively different results are obtained from a reporter assay compared with a cell-specific assay. For example, analog **29**is much more effective than **46**and **48**in preventing TPA-induced COX-2 expression (figure 6) whereas **29**is less active than **46**and **48**in a reporter assay (Table 1). Studies are underway to address this issue of screening in a cell-specific manner and evaluating the usefulness of reporter assays.

## Conclusion

Substituted *trans*-stilbenes represent a class of compounds that can inhibit or enhance the TPA-induced activation of AP-1. Small changes in structure can convert an inhibitor into an enhancer. The ability of *trans*-stilbenes to inhibit or enhance the effects of TPA does not depend upon their anti-oxidant properties.

## Abbreviations

AP-1: activator protein-1; TPA: 12-O-tetradecanoylphorbol-13-acetate; TRE: TPA-responsive element.

## Authors' contributions

LMD directed the synthetic chemistry and helped design the study; LAH and AMG collected the data related to the effects of substituted *trans*-stilbenes on TPA-induced activation of AP-1; RAO designed and directed studies involving use of macrophage cells; and DLVJ helped design the study and wrote the manuscript.
